# What viruses tell us about evolution and immunity: beyond Darwin?

**DOI:** 10.1111/nyas.14097

**Published:** 2019-04-29

**Authors:** Felix Broecker, Karin Moelling

**Affiliations:** ^1^ Department of Microbiology Icahn School of Medicine at Mount Sinai New York New York; ^2^ Institute of Medical Microbiology University of Zurich Zurich Switzerland; ^3^ Max Planck Institute for Molecular Genetics Berlin Germany

**Keywords:** Darwin, viruses, evolution, horizontal gene transfer

## Abstract

We describe mechanisms of genetic innovation mediated by viruses and related elements that, during evolution, caused major genetic changes beyond what was anticipated by Charles Darwin. Viruses and related elements introduced genetic information and have shaped the genomes and immune systems of all cellular life forms. None of these mechanisms contradict Darwin's theory of evolution but extend it by means of sequence information that has recently become available. Not only do small increments of genetic information contribute to evolution, but also do major events such as infection by viruses or bacteria, which can supply new genetic information to a host by horizontal gene transfer. Thereby, viruses and virus‐like elements act as major drivers of evolution.

## Graphical abstract

Here, we describe mechanisms of genetic innovation mediated by viruses and related elements, which during evolution caused major genetic changes beyond what was anticipated by Charles Darwin.

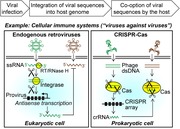

## Beyond Darwin?

Charles Darwin revolutionized our understanding of the origin and evolution of species. The central tenets of his theory of evolution state that hereditary variation occurs slowly due to the gradual accumulation of random small modifications that are subject to natural selection. The discovery of DNA as a carrier of the hereditary information then allowed linking inherited traits to mutations in DNA. Today, we also know that RNA, with its high plasticity due to the relatively high infidelity of RNA polymerases, can exert strong evolutionary influences. New technologies for high‐throughput genome sequencing have recently led to the discovery of multiple novel forms of mutations and genetic alterations.

Fundamentally new insights into these influences have come from viruses and microorganisms, infectious agents that can supply genetic material into recipient cells by horizontal gene transfer (HGT). For instance, up to two‐thirds of the human genome sequence is derived from viruses and transposable elements,^1^, ^2^ and a smaller proportion of DNA sequence originates from bacteria and other microorganisms.^3^, ^4^, ^5^ In contrast to point mutations, small insertions, or small deletions, which have conventionally been regarded as the major driving forces of evolution, the supply of new genes by HGT can cause dramatic changes to an organism almost instantaneously. In addition to HGT, major genetic changes are induced by crossover and recombination events, (retro)transposition activity, transformation, and conjugation;^3^, ^4^, ^5^, ^6^ well‐studied examples include uptake of plasmids that confer antibiotic resistance to a bacterial cell.^7^ Furthermore, RNA agents, including RNA viruses and viroids, typically lack proofreading mechanisms during their replication and thus can accumulate mutations more rapidly than replicating DNAs.^8^, ^9^ Retroviruses, with their high genomic plasticity and life cycle that includes obligatory integration into the host genome, are among the major drivers of evolution by, among other things, providing novel genes and regulatory elements.^10^, ^11^, ^12^, ^13^ Retroviruses have accumulated in large numbers into eukaryotic genomes, mediate HGT, transduce cellular genes including oncogenes, and contribute to cancer.^1^, ^2^, ^10^

Genetic transfer events between different species across all domains of the tree of life, and cross‐talk between various genomes (cellular and viral), raise new questions on the definition of a species.^14^ The conventional definition of a species assumes that any organism acquires its set of genes solely from its parents (subject to minor variations in each generation due to imperfect copying mechanisms). However, mechanisms like HGT can introduce genetic material even from distantly related species, including viruses or smaller genetic agents such as transposable elements.^14^

Microbial and viral infections, furthermore, can lead to endosymbiotic relationships and are at the origin of eukaryotic structures such as mitochondria and chloroplasts (both originating from formerly free‐living bacteria), and possibly the nucleus, which may have originated from a complex virus.^15^, ^16^, ^17^ Viral infections were crucial for the evolution of the mammalian placenta^11^ and various immune systems in prokaryotes and eukaryotes,^18^, ^19^, ^20^ and they shaped genomes throughout evolution—genomes that now serve as archives of previous viral infections.^21^

Single‐stranded RNA (ssRNA) is the most innovative biomolecule known. We can learn about its properties by analyzing ribozymes (catalytic RNA species) and closely related virus‐like viroids. An entire living world could be built from RNA only, whereby RNA could replicate, cleave, join, form peptide bonds, and evolve depending on environmental changes.^22^, ^23^ Evolutionary innovations were then fixed in the form of a more stable and less variable molecule, double‐stranded DNA (dsDNA), which likely arose after RNA. The known DNA polymerases typically introduce fewer mutations than do RNA polymerases, due to efficient proofreading mechanisms.^8^, ^9^ DNA, however, can also change significantly by mechanisms such as mobility of transposable elements (DNA transposons and retrotransposons). Transposable elements can be regarded as capsid‐free viruses^24^ that are trapped within host cells but occasionally can be transferred between different cellular organisms.^25^, ^26^

## The evolutionary tree of life

**Figure 1:**
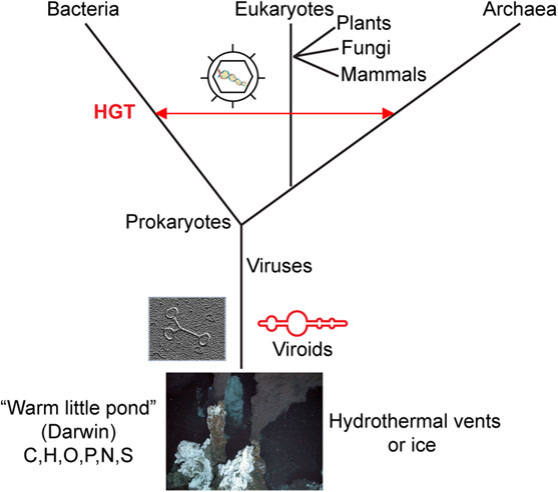
Phylogenetic tree based on ribosomal DNA sequences^27^ (not to scale). The red line indicates horizontal gene transfer (HGT) by viruses and related elements.

A simplified tree of life is shown in Figure 1. The three arms represent phylogenetic relationships between the bacterial, archaeal, and eukaryotic domains, as inferred by ribosomal DNA sequences.^27^ The horizontal line symbolizes HGT events among the three arms. HGT has been a frequent phenomenon during evolution and is often mediated by viruses and other selfish genetic elements.^28^, ^29^, ^30^, ^31^ For example, the human genome contains up to two‐thirds of sequences originating from retroviruses and transposable elements, and at least 145 genes (of a total of 21,000) are likely derived from bacteria, archaea, fungi, protists, and plants.^1^, ^2^, ^5^ Viruses infect organisms of all three domains of cellular life “from root to the leaves,” but due to their polyphyletic nature are often not included in the tree of life.^32^ We, however, hypothesize viruses to be at the root.^33^ This is speculative but based on the fact that virus‐like elements, such as viroids, are the smallest known replicating entities with structural and not coding information. Catalytically‐active and replicating species can arise from populations of RNA with random sequences or quasispecies.^23^ Furthermore, the recently discovered giant viruses indicate that the evolutionary transition from virus to cell may be a continuum;^34^ and ribosomes have likely evolved from simple ribozymes in the ancient RNA world.^35^ Thus, although there are opposing views,^36^ we^21^, ^33^ and others^37^, ^38^ think that viruses must be located far down at the root of the tree of life.

Bacteria, archaea, and eukaryotes are normally described as originating from the last universal common ancestor, which requires DNA, protein synthesis, and must, therefore, have evolved for long periods from simpler forms of life. The origin of life on Earth, in general, is speculative but most likely included forms of noncoding replicating RNA‐like molecules as the first biopolymers, possibly linked with phosphodiester bridges or more stable peptide linkages as peptide nucleic acid.^39^, ^40^ In the test tube, catalytically active RNA molecules have been selected from a pool of ∼10^15^ RNA polymers 220 nt in length with random sequences that can adopt a multitude of conformations, including ribozymes and tRNA‐like structures described as quasispecies.^23^, ^41^, ^42^ Ribozymes do not encode proteins but contain structural information through hairpin−loop folds, can perform a multitude of catalytic reactions, including RNA cleavage and ligation, as well as peptide bond formation; they can mutate and thereby adjust to environmental changes and replicate without the help of protein‐based polymerases.^22^, ^23^

**Figure 2:**
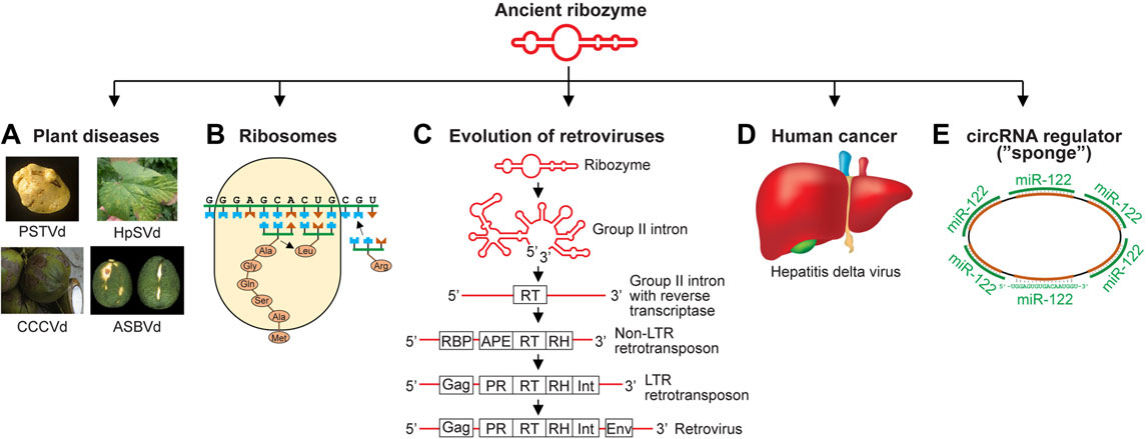
Simple ribozymes evolved into various structures. (A) Viroids are related to ribozymes, some have retained ribozyme activity. PSTVd, Potato spindle tuber viroid; HpSVd, Hop stunt viroid; CCCVd, Coconut cadang‐cadang viroid; ASVBd, Avocado sunblotch viroid. (B) A ribozyme became the catalytically active center of ribosomes.^47^ (C) Ribozymes may have evolved gradually to today's retroviruses by acquiring coding information.^50^ APE, apurinic endonuclease; Gag, group‐specific protein; Env, envelope; Int, integrase; PR, protease; RBP, RNA‐binding protein; RH, RNase H; RT, reverse transcriptase. (D) The hepatitis delta virus likely originated from a plant viroid that picked up coding information from a cell.^51^ (E) Circular RNAs (circRNAs) are involved in gene regulation by binding to short regulatory RNAs comparable to a “sponge” and have been identified in all domains of life, suggesting an ancient evolutionary origin.^54^, ^55^

Ribozymes are closely related to viroids, circular naked RNA virus‐like elements that can cause diseases in plants, such as the Potato spindle tuber viroid (Fig. 2A).^43^, ^44^ Some plant viroids found today have retained ribozyme activity, but many have lost it; they are replicated by RNA polymerases of the host cell. Loss of function is an often‐underestimated evolutionary force facilitated by the supply of functions through the environment, causing a reduction of genome size to reduce the energy required for replication.

The first ribozymes may have formed at hydrothermal vents at the bottom of the oceans in niches with large surface areas and concentrating effects where minerals supply metal ions as catalysts and high temperatures accelerate chemical reactions.^45^ However, even ice can support ribozyme activity, as the progressive dehydration during ice crystal formation concentrates the substrates (ribonucleotide triphosphates and magnesium salts), while ribozyme activity is retained even below 0 °C.^46^ Later, during cellular evolution, ribozymes became integral parts of ribosomes and constitute the enzymatically active component for protein synthesis by forming peptide bonds: “Ribosomes are ribozymes” (Fig. 2B).^47^ There are examples of ribozymes that acquired coding information during evolution. For example, it has been suggested that a reverse transcriptase gene became inserted into group II introns,^48^, ^49^ which then may have further evolved into retroviruses^50^ (Fig. 2C).

A rare case of a circular RNA (circRNA) with coding capacity is the hepatitis delta virus (HDV), which presumably originated from a noncoding plant viroid. HDV integrated a gene, likely of cellular origin, into its catalytic ribozyme (Fig. 2D).^51^ HDV contributes to liver cancer in combination with hepatitis B virus, which incorporates HDV into its virions to allow for transmission to other cells and organisms.^51^, ^52^ Another viroid‐like RNA with protein‐coding information is the smallest known naturally occurring replicating RNA only 220 nt in length, the small circular satellite of rice yellow mottle virus (scRYMV) that is made up completely of coding sequences.^53^ The origin of the protein sequences is unclear. scRYMV can be considered as an evolutionary intermediate from simple ribozymes toward coding RNAs.

Recently, circRNAs have been described as molecules involved in mammalian gene regulation by binding to a number of short regulatory RNAs comparable to an absorbing “sponge” (Fig. 2E).^54^ circRNAs have subsequently been identified in all domains of cellular life,^55^ suggesting an evolutionary ancient origin. Alternatively, modern circRNAs may have several independent origins, yet all are based on noncoding RNA (ncRNA).

**Figure 3:**
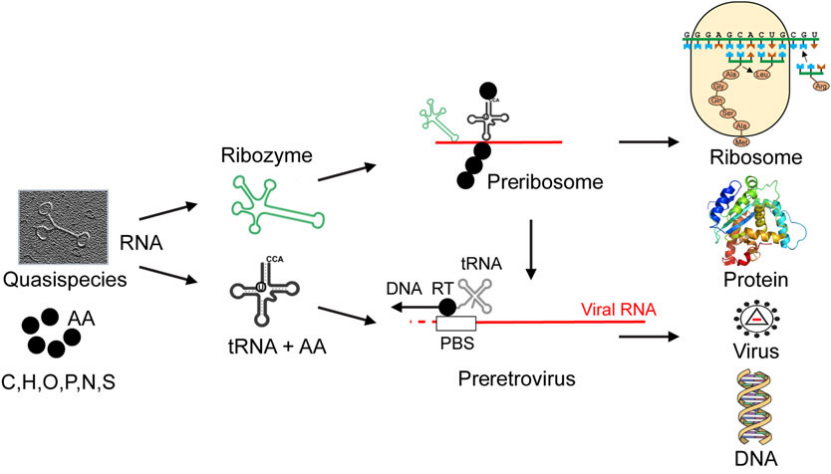
Hypothetical models for the evolution from ribozyme to ribosomes and retroviruses.

tRNA‐like folds may have been among the earliest RNA structures together with ribozymes and have played a central role in the evolution of replication and protein synthesis (Fig. 3).^56^, ^57^ Originally, tRNA‐like structures likely evolved as a tag to initiate RNA genome replication in the RNA world before the advent of proteins.^56^ This functionality can still be observed in some contemporary viruses where tRNAs and tRNA‐like structures are essential for replication.^58^ For example, a number of plant positive‐strand RNA viruses bear tRNA‐like structures carrying one specific amino acid at the 3’‐termini, which—among other functions—act as primers to initiate minus‐strand RNA synthesis by the viral replicase.^58^ Cellular tRNAs also act as primers for the reverse transcriptase of retroviruses, plant pararetroviruses, and LTR retrotransposons, to initiate DNA synthesis.^59^

tRNAs may have also played a role in the formation of early retrovirus‐like structures, selecting for a tRNA‐binding site on evolving RNAs. This combination is maintained in extant retroviruses where tRNAs serve as primers to initiate replication *via* a DNA copy. In addition, precursor tRNAs likely evolved into ribosomal RNAs which bear many striking similarities to tRNA sequences.^50^, ^60^ In present‐day cellular organisms, tRNAs serve as the carriers of amino acids to the ribosome to enable protein synthesis. Highlighting the versatility of the tRNA fold, other functions have also been described. For example, tRNA‐derived small RNAs have been shown to regulate gene expression and suppress transposable elements in eukaryotes.^61^, ^62^ In addition, tRNAs are considered as a relevant component in transgenerational inheritance through germline cells (see below).

It may be interesting to note that not only ribozymes but also deoxyribozymes likely evolved before the emergence of a reverse transcriptase, through chemical removal of oxygen from ribonucleotides.^23^

## Viruses as drivers of evolution

Influenza viruses, whose negative‐sense ssRNA genomes are segmented into eight parts that are copackaged into the virion, provide a good example for two types of evolutionary processes. On the one hand, the surface glycoproteins, subject to immune pressure by the host, undergo incremental point mutations (as proposed by Darwin), a process termed antigenic drift.^63^ On the other hand, entire gene segments can be exchanged if a cell is coinfected with two or more different influenza viruses, a process called antigenic shift. Antigenic drift causes subtle antigenic alterations that help the virus to gradually evade host immunity. Antigenic shift, on the other hand, can instantaneously cause a drastic antigenic change. The introduced glycoproteins may be entirely unknown to the immune systems of large parts of the population. Therefore, antigenic shift events (and not antigenic drift) were at the origin of the major influenza virus pandemics such as the 2009 H1N1 “swine flu,” a triple reassortant virus containing genome segments originating from human, swine, and avian viruses.^64^ Influenza viruses were among the first to indicate the difference between point mutations and gene exchange.^65^ Antigenic shift thereby is a form of HGT between related influenza viruses.

**Figure 4:**
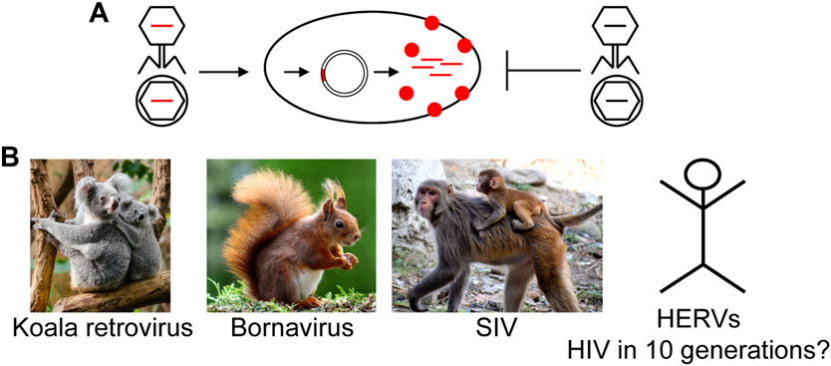
Endogenization of viruses causes immunity. (A) Schematic representation of superinfection exclusion. A phage/virus (genomes indicated as red lines) infects a cell and the genome is integrated into the cellular genome. RNA or proteins from the endogenized phage/virus protect against superinfections by related invaders (genomes shown as black lines). (B) Examples for superinfection exclusion include koala retrovirus,^74^, ^75^, ^76^ bornaviruses in ground squirrels,^94^ and simian immunodeficiency virus (SIV).^77^ In humans, human endogenous retroviruses (HERVs) may cause resistance against related retroviruses including HIV.^153^ In addition, it has been suggested that endogenization of HIV itself can happen^80^ and may cause resistance to AIDS in the future.

Retroviruses can also undergo subtle mutational changes that are facilitated by the infidelity of their reverse transcriptase.^10^, ^65^ This helps the virus to efficiently escape the host immunity. More drastic genetic changes can result from recombination events with cellular genes, including oncogenes, as described below. Each retroviral infection, following reverse transcription of the ssRNA genome into a dsDNA provirus, supplies the host cell's genome with a set of new genes, *gag*, *pol*, and *env* and auxiliary genes, as in the case of lentiviruses like HIV.^10^, ^65^ Retroviral insertion can induce downstream activation of cellular genes by the retroviral promoter, or insertional inactivation.^13^, ^66^, ^67^ Such events are a potential cause of chronic diseases, including cancer. For example, the expression of the *c‐Myc* proto‐oncogene can be dysregulated through the nearby insertion of avian leukosis virus, leading to B cell lymphomas in chickens.^68^ Infections of somatic cells only affect one individual since the integrated virus is not passed on to the next generation. Some retroviruses, however, can integrate into germline cells and are consequently inherited by future generations. These are the endogenous retroviruses that populate eukaryotic genomes.^65^ Endogenous retroviruses can accumulate to high numbers in genomes through reinfection and/or intracellular amplification.^1^, ^10^, ^12^ In rare cases, other RNA viruses, such as Bornaviruses, and DNA viruses can be integrated (see below and Fig. 4). Subsequently, viral genes can be captured by the host cell to exert novel functions, as described below for the syncytins.

## Oncogenes and cancer

Retroviruses are potent mediators of HGT, which was initially discovered by oncogenes that are taken up by recombination between the integrated viral genome and cellular genes.^65^ Many oncogenes were selected for by their strong growth promoting ability *in vitro* and a high potential for malignant transformation or tumor growth in animal models.^10^, ^69^, ^70^ Some of the products of oncogenes, such as ErbB, Ras, Raf, or Myc, are dysregulated in human cancers and are targets for anticancer therapy.^70^ The infidelity of retroviral replication by the error‐prone reverse transcriptase often leads to mutations, such that cellular and viral oncogenes can differ. For example, the *v‐Src* oncogene of Rous sarcoma virus (RSV) is a C‐terminally truncated variant of the cellular *c‐Src* proto‐oncogene. The truncation eliminates a regulatory domain such that the v‐Src protein causes malignant transformation, whereas c‐Src with the intact regulatory domain does not.^71^ Thus, RSV serves as a vehicle for HGT, carrying a mutated variant of the *c‐Src* gene to other cells.

Oncogenes are often taken up by retroviruses at the expense of replicating genes, which leads to nonreplicating defective viral entities that require the supply of the lacking genes of other superinfecting retroviruses *in trans* to infect other cells.^10^, ^21^, ^69^ The generally modular structure of viral genomes reflects how they have evolved by gene uptake from various sources.^72^ This allows for *de novo* recombination of genes occurring naturally or artificially by genetic engineering, which is the basis for the design of viral gene therapy vectors carrying therapeutic genes, or tailor‐made phages for antibacterial purposes.^73^

## Endogenous viruses

The process of endogenization has been studied recently in koalas where an ongoing endogenization of a retrovirus into the germline is observed in real time (Fig. 4).^74^, ^75^, ^76^ In the early 1900s, some koalas were isolated on islands off the Australian coast as they were endangered to go extinct. The animals on mainland Australia (and some of the islands) were infected via a cross‐species transmission with a virus related to gibbon ape leukemia virus.^74^, ^75^, ^76^ Many animals succumbed to the virus termed koala retrovirus (KoRV). However, due to its capacity to infect germline cells, KoRV became endogenized in some koalas.^74^, ^75^, ^76^ The endogenized viruses likely protect the animals against *de novo* infections with exogenous KoRV.^74^, ^75^ Since koala populations on some islands remained free of KoRV, it is likely that the first endogenization event has occurred within about the last 100 years.^74^ Endogenization is still ongoing as a significant degree of interindividual variability is observed with respect to numbers and integration sites of KoRV elements.^74^ KoRV is an example for an integrated retrovirus that may protect against *de novo* infection by similar viruses, a mechanism designated as superinfection exclusion that has been described for other retroviruses, and also nonretroviruses, as well.^18^, ^50^ Superinfection exclusion can be based, for instance, on destruction of the invading viral genes by RNase H‐like nucleases or the expression of viral gene products that block cell surface receptors.

Protection by a virus against superinfecting viruses is presumably also the case in some monkey species that allow for replication of simian immunodeficiency virus (SIV), a close relative of HIV, but do not succumb to the infection.^77^ These monkeys are presumably resistant because their immune systems were shaped by lentiviruses such that they became resistant to SIV‐induced immunodeficiency.^77^ One can speculate that this may also happen with HIV in humans in the future, which will likely take at least 10 generations, as judged from the koalas, whose generation time is about 7.8 years. HIV has indeed been shown to be able to infect germline cells, which would be a requirement for endogenization into the germline.^78^, ^79^ In addition, there is evidence that defective HIV proviruses protect human T cells from superinfection with HIV.^80^

During evolution, retroviral endogenization happened frequently enough to populate the human genome with 450,000 retroviral elements that have typically accumulated inactivating mutations if the expression of viral genes was not selected for. The human genome consists of almost 50% of full‐length or truncated endogenous retroviruses and related retroelements.^1^ Some human genes, like protein kinase inhibitor beta (*PKIB*), consist of up to 80% of such elements, usually located in introns.^81^ This raises the question about what the upper limit of retrovirus‐related insertions may be in eukaryotic genomes. The majority of endogenous retroviruses have been degenerated by homologous recombination of the long terminal repeats (LTRs) and elimination of the internal sequences.^1^, ^13^ These solitary LTRs are so abundant that the total genetic information would be longer than the human genome itself if they were full‐length retroviruses of about 10 kilobases.

As the proof that the ancient retroviral insertions in the human genome are derived from formerly infectious retroviruses, a consensus sequence from the sequences of nine full‐length endogenized retroviruses was constructed.^82^ The sequence was converted into a synthetic virus, designated as Phoenix, which was able to form virus particles and infect mammalian cells *in vitro*.^82^

A major benefit for human ancestors was germline infections with two retroviruses now designated as human endogenous retrovirus (HERV) types W and FRD.^83^, ^84^ The corresponding infectious retroviruses entered the ancestral germline about 30 and 45 million years ago, respectively. Gene products of these viruses, syncytins, which are derived from the retroviral envelope protein and related to HIV gp41, are involved in placental development.^11^ Specifically, the coordinated expression of syncytins 1 and 2 is required for the formation of the syncytiotrophoblast that establishes the interface between maternal blood and embryonic extracellular fluid. In addition, syncytins mediate the protection of the fetus from maternal immune rejection through the immunosuppressive domain.^85^ Similarly, HIV has the ability to fuse cells via its gp41 protein, which leads to syncytia formation by cell fusion. In the case of syncytins, ancient retroviruses induce immune suppression for the benefit of the host.

Other than providing novel genes to the host, endogenous retroviruses and other retroelements have been shown to regulate host gene expression by providing promoters, enhancers, transcription factor binding sites, and other regulatory elements. We have shown that some LTRs of the HERV‐K(HML‐10) family of HERVs, which invaded the ancestral genome about 35 million years ago, have retained promoter activity.^13^ Moreover, one of the LTRs primes a regulatory transcript that suppresses the expression of the death‐associated protein‐3 gene (*DAP3*) implicated in apoptosis.^13^ Knockdown of the retroviral transcript was sufficient to induce apoptosis in cancer cell lines *in vitro*, suggesting that the LTR may contribute to the apoptosis‐evading phenotype of some cancer cells. However, a single endogenous retrovirus is unlikely a major cause of cancer; rather it may contribute to the phenotype of specific cancerous cells. Similar regulatory transcripts originating from other LTRs in the human genome likely contribute to the complex network of human gene regulation and possibly to cancer.^13^ In addition, endogenous retroviruses have been shown to provide regulatory elements including transcription factor binding sites to mammalian genes, which, for example, have been shown to modulate innate immunity pathways.^12^

Recently, HERV‐K has been suspected to contribute to various human diseases, such as cancer and amyotrophic lateral sclerosis,^86^ and reverse transcriptase activity, likely of retroelement origin, to Alzheimer's disease (AD)^87^ (see below).

In addition to endogenous retroviruses, mammalian genomes harbor sequences derived from *Circoviridae*, *Filoviridae*, *Bornaviridae*, *Parvoviridae*, *Herpesviridae*, and others.^88^, ^89^, ^90^, ^91^, ^92^ A well‐studied example of the function of endogenous nonretroviral sequences are Borna disease viruses (BDVs), negative‐sense ssRNA viruses that cause fatal encephalitis in sheep, horses, and cattle.^88^, ^93^ Horses, for instance, are susceptible to Bornaviral disease, which includes signs of depression. Intriguingly, these susceptible species have no detectable BDV‐related sequences in their genomes.^88^ In contrast, less susceptible species, including primates, mice, and rats, have genomic BDV sequences.^88^ Indeed, experimental evidence for a protective role of endogenous BDV sequences against BDV infection has been obtained in squirrels.^94^ Various other virus‐derived sequences in the genomes of mammals and other species have been recruited for antiviral and immune‐related functions, as described elsewhere.^12^, ^18^, ^19^, ^20^

Noncoding RNA in the human genome amounts to about 98% of the sequence, leaving only about 2% for protein‐coding information.^1^ In contrast, it is low or close to zero in viruses or prokaryotes.^95^ In RNA viruses, the 5´‐termini are often noncoding with structural, not genetic information, such as dimerization signals in retroviral genomes, the transactivation response element in HIV or internal ribosomal entry sites found in many positive‐sense ssRNA viruses.^65^

## Transposable elements

Related to viruses are transposable elements found in pro‐ and eukaryotic genomes.^24^ The transposable elements are either DNA transposons exerting a cut‐and‐paste transposition or retroelements, which undergo reverse transcription and integration at a new genomic locus by a copy‐and‐paste mechanism that leads to gene duplication—a major property of complex genomes.^1^, ^96^ Gene duplication is a major innovative step since one copy can maintain its functions, while the other one can change. Transpositions can cause major changes in the host's genome, and may compromise its integrity, as integrations are mutagenic events.^67^ Highly abundant retrotransposons are indicators of active evolution and gain of functions of an organism which has to cope with new environmental conditions. This seems to be the case in marine ecosystems, as retrotransposon activity of ocean plankton is apparently high, with reverse transcriptase genes amounting to up to 13.5% of the metagenomes.^97^ Also, RNase H‐like genes are of high frequency in marine plankton, with about 15 copies per cell on average.^50^ Evolutionary events mediated by transposable elements appear to be constantly ongoing in the oceans and remain to be studied in detail.^97^

Another measure for the activity of transposable elements is the expression level of transposases.^98^ Transposases can introduce innovation in bacterial genomes and thereby promote adaptation to dynamic environments. They not only influence the host cell's genome, but can also mediate HGT between cells.^99^ In marine bacteria, transposase expression levels are particularly high under environmental stress such as low oxygen as well as in biofilms and particles.^98^ This may relate to higher rates of genome modifications and HGT necessary to adapt to environmental stress, whereby dense populations may provide hotspots of bacterial evolution.^98^

In eukaryotes, transposon activity is counteracted by RNA interference (RNAi).^100^ In stem cells and sperm cells of various species, such as mice or *Caenorhabditis elegans*, and likely also in humans, transposon activity is silenced by a dedicated RNAi mechanism, the Piwi‐interacting RNA (piRNA) pathway.^101^ Piwi proteins mediate the destruction of RNA from transposable elements, guided by piRNAs, 26−31 nt in length. They are part of the Argonaute protein family, which have the same three‐dimensional structure as the retroviral RNase H.^102^ This silencing mechanism suppresses transposon activity in stem and germline cells, where too high transposon activity may result in the accumulation of detrimental genetic defects; too much “jumping” would result in an “error catastrophe,” as defined by Manfred Eigen, leading to cell death.^41^, ^103^, ^104^ Inactivation of Piwi activity can lead to male infertility, highlighting the importance of transposable element silencing.^105^ The frequency of piRNAs as mediators of transgenerational inheritance transmitted *via* sperm cells is presently a matter of debate.^106^

## Related immune mechanisms in eukaryotes and prokaryotes

**Figure 5:**
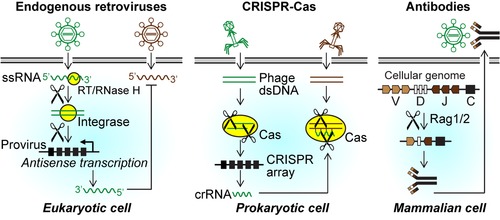
Endogenization of retroviruses and phages into the host genomes to serve as antiviral defense is orthologous in pro‐ and eukaryotes (crRNA stands for CRISPR RNA). The generation of diversity of antibodies by V(D)J recombination also involves virus‐like elements, *Transib* transposons.^111^

Cellular organisms have evolved a plethora of antiviral and antiphage defense systems.^107^, ^108^ It is worth comparing antiviral mechanisms mediated by viruses in eukaryotes (“viruses protect against viruses”)^12^, ^18^, ^19^, ^20^, ^109^ with the defense systems of prokaryotes where “phages protect against phages.”^18^, ^110^ Many mechanisms are analogous and can be described as the endogenization of invading sequences to defend related invaders. In this regard, HERV‐mediated protection and CRISPR/Cas9 are analogous.^18^ Also, superinfection exclusion and viral interference are related defense mechanisms found in both eukaryotes and prokaryotes.^18^ A central role in many cellular immune systems is played by RNase H‐like proteins, such as the Cas9 nuclease in CRISPR/Cas9, the Piwi domain in Argonaute‐based silencing, and the RAG1 recombinase that mediates V(D)J recombination to diversify antibody and T cell receptor sequences (Fig. 5).^18^, ^81^, ^111^

RNases H are typically not sequence specific, but their cleavage sites depend on fused domains, which in the case of HIV is the reverse transcriptase, together with structural properties of the polypurine tract, a preferred binding site in the RNA template.^112^ This specificity creates the dinucleotide overhangs essential for subsequent dsDNA provirus integration by the integrase, which also exhibits an RNase H–fold.^113^ The Cas9 enzyme of CRISPR/Cas9 is an RNase H−related enzyme directed by a guide RNA to cleave the DNA strand in RNA−DNA hybrids, as in this case defense is directed against an invading phage DNA.^114^ RNases H can even cleave an RNA strand in dsRNA, highlighting the versatility of this protein fold.^115^

In addition, the components of the host antiviral defense systems, in prokaryotes or eukaryotes, such as RNAi‐type defense, are similar to the set of gene products that make up retroviruses.^81^, ^109^ These similarities are, among others, the lengths of the cleavage products of around 20 nt, staggered cleavage events with dinucleotide overhangs, the common fold of Piwi and RNase H domains, nucleic acid binding by Argonaute and reverse transcriptase, unwinding and helicase activities, Dicer and integrase, and caspase and protease; all of these are orthologues, as described.^81^, ^109^

Antiviral responses can be based not only on cleavage enzymes guided by nucleic acids, but also on blocking infection through receptor occupation by viral gene products as well as transcriptional or translational inhibition.^18^ Viruses and genetic parasites as inventors of antiviral systems have also been described by Villarreal.^19^, ^20^

The reverse transcriptase, first discovered in retroviruses, is highly abundant in almost all cellular species. There are more than a thousand bacterial variants with mostly unknown functions and reverse transcriptases are also abundantly expressed in marine plankton.^97^, ^116^ Reverse transcriptase activity in the human brain may play a role in the development of AD, affecting the amyloid precursor protein gene (*APP*).^87^ Recombination of the gene with different reverse transcribed “genomic cDNAs” of various full‐length or truncated spliced mRNAs was more frequently found in the brains of AD patients than in healthy controls.^87^ The genomic cDNAs resemble retrotransposition events and pseudogene formation by long interspersed nuclear elements (LINEs) that encode a reverse transcriptase.^87^ Indeed, LINE‐1 retrotransposition events have been detected in the human brain, creating a mosaicism of somatic *de novo* LINE‐1 insertions.^117^ It has been suggested that this mosaicism may have evolved to increase the complexity and diversity of the mammalian brain.^118^

The reverse transcriptase can also be part of diversity‐generating retroelements (DGRs) that are found in bacterial and phage genomes.^119^ DGRs utilize the infidelity of the reverse transcriptase to diversify DNA sequences. The prototype DGR of *Bordetella* bacteriophage BPP‐1 is able to modify the phage's receptor specificity and thereby alter tropism.^119^

## Epigenetics and paramutations

**Figure 6:**
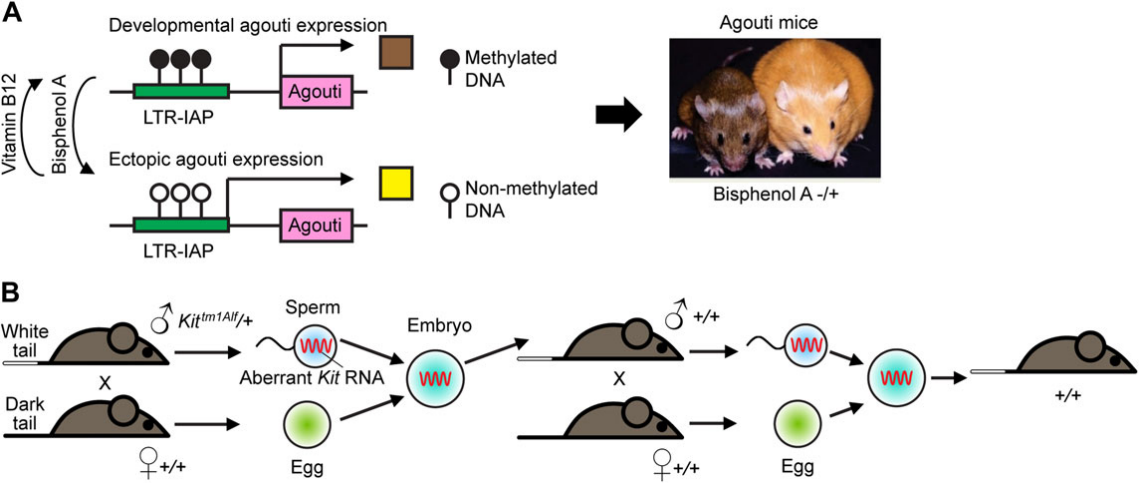
Epigenetics and paramutations as examples of non‐Mendelian inheritance. (A) Environmental effects on fur color of *agouti* mice by vitamin B12 or bisphenol A on methylation of the alternative long terminal repeat (LTR) promotor of an intracisternal A particle (IAP), epigenetic modifications that are inheritable.^121^, ^122^ (B) Transgenerational regulation of expression of *Kit* by RNA‐mediated inheritance *via* sperm cells.^132^ Heterozygous male mice with a *Kit^tm1Alf^* allele (a null allele that makes no functional Kit protein but an aberrant *Kit* RNA; a “paramutagenic” allele) and a normal wild‐type (+) allele have a tail with a white tip. The aberrant *Kit* RNA is packaged into sperm cells and transmitted to the embryo. Progeny carry a wild‐type *Kit* allele transmitted by the father, but action of the transmitted aberrant *Kit* RNA still gives rise to the white tail. This allows for paramutation of the wild‐type *Kit* allele and further transmission of the white tail. Loss of aberrant RNA through dilution over successive generations might lead to gradual loss of the paramutation.

One of the mechanisms of mammalian organisms to control the activity of endogenous retroviruses is epigenetic silencing of the LTRs.^120^ In some cases, such epigenetic modifications have been recruited to regulate host genes. These can cause major changes in phenotypes beyond Darwin. A well‐studied animal model for epigenetic effects is the inbred agouti mouse in which *agouti* expression depends on the degree of methylation of an alternative promoter located in the LTR of an intracisternal A particle (IAP), a type of endogenous retrovirus.^121^ Hypomethylation of the LTR leads to strong *agouti* expression, causing mice to have yellow fur and to develop obesity (Fig. 6). Hypermethylation prevents *agouti* expression, resulting in brown, nonobese mice. The diet influences methylation of the LTR and thereby the mouse phenotype. Obese yellow mice can become lean brown mice by supplementing their diet with methyl donors, such as vitamin B12 or folic acid.^122^ In contrast, the toxin bisphenol A causes LTR hypomethylation; exposed mice become yellow and obese.^121^ Another gene susceptible to the level of methylation of an IAP is the *axin fused* allele that influences tail kinking in mice.^123^ The total number of IAP elements in the mouse genome is about 1000. About 100 of them have been identified recently as being variably methylated in C57BL/6 mice, they might, therefore, be involved in epigenetic regulation of gene expression, similar to *agouti* and *axin fused*.^124^, ^125^ In germline cells, epigenetic marks are normally erased and remodeled in a process termed *epigenetic reprogramming*.^120^ Therefore, epigenetic changes are mostly transient and only affect one individual in its lifetime. However, in some cases, modifications may escape eradication and they can be passed on to the offspring, whereby the altered epigenetic modifications typically return to the normal state within a few generations by dilution.^126^ For example, the epigenetic marks regulating *agouti, axin fused*, and a third locus have been shown to be transgenerationally inheritable.^121^, ^123^, ^124^ Epigenetic inheritance not directly mediated by DNA modifications but by small RNAs, termed paramutations, is presumably more stable over time.^127^ Small RNA‐mediated paramutations (see below) can last for at least 20 generations in the nematode *C. elegans*.^128^ Recently, tRNAs and R‐loop formation have also been found as mechanisms that contribute to epigenetic changes of gene expression.^129^, ^130^

The phenomenon of paramutations was discovered in maize by the Canadian plant geneticist Royal Alexander Brink in the 1960s.^131^ Paramutations do not follow Mendelian laws and are, in contrast to transient changes, nongenetic modifications that are inherited and long‐lasting over multiple generations.^132^ Paramutations have recently been attributed in some cases to various RNAs species, including piRNAs, miRNAs, tRNA‐derived small RNAs, long ncRNAs, and others, which are transmitted through the sperm of mice.^132^, ^133^, ^134^, ^135^, ^136^, ^137^ Transgenerational inheritance was shown, for instance, to have an impact on the tail phenotype of mice and was identified as being mediated by miRNAs specific for the tyrosine kinase proto‐oncogene *Kit*, transmitted through the sperm.^132^ Similarly, in mice, cardiac hypertrophy,^133^ body size,^134^ obesity,^135^ metabolic syndrome,^127^ and stress‐induced gene expression programs^136^, ^137^ were found to be influenced by transgenerational inheritance mediated by miRNAs, with the phenotypic effects usually lasting over several generations. It has recently been suggested, however, that transgenerational inheritance in mammals may be a relatively rare phenomenon.^106^

## Endosymbiosis

Endosymbiosis, or symbiogenesis, is another phenomenon where major genetic innovation can occur through a single event. Symbiogenesis is defined by the introduction of new genetic information by uptake of an entire organism, whereby the symbiotic relationship is extended beyond a “master and slave” interaction.^138^ Symbiotic relationships between partners with bacteria and viruses have been important events during evolution,^138^ including flowering plants.^139^, ^140^ The uptake of bacteria leading to the evolution of chloroplasts and mitochondria, for example, are considered unique events during eukaryotic evolution. The engulfed bacteria lost or delegated the majority of genes, around 3000, to the host cell nucleus such that the human mitochondria, for instance, only retained 37 genes that are specialized to energy production.^141^, ^142^ Mitochondria likely originated from *Rickettsiales* bacteria^143^ and chloroplasts are related to cyanobacteria.^144^ These events, however, are not as rare as thought and similar uptakes of bacteria into other cells may still happen today. For example, bacteria of the *Rickettsia* genus lost the ability for autonomous replication and became dependent on the host.^145^ Thus, genomes do not only increase in size during evolution, but there are also counteracting forces, loss of genes as exemplified by endosymbionts and *Rickettsia*. This may be an energy‐saving measure of symbionts compared to autonomously living species. The endosymbiotic *Wolbachia* spp. bacteria can even transfer genes into their arthropod hosts and influence host reproduction and speciation.^146^ In addition, the nucleus, a hallmark of eukaryotic cells, may originate from a giant virus related to poxviruses or mimiviruses.^15^, ^16^, ^17^ Giant viruses exclusively replicate in the cytoplasm and may have been autonomous entities originally,^24^ similar to the precursors of mitochondria and other plastids. Further supporting evidence for viral nucleogenesis comes from phenotypic similarities of viral factories (the site of replication of giant viruses in cellular cytoplasm) with the nucleus.^17^ The nucleus may have emerged by fusion of the cellular genome with the giant virus genome, which then became the nucleus, losing the ability for virus production.^17^

The mechanism of endosymbiosis indicates that replicating entities can give up an autonomous lifestyle and become parasitic within a richer environment, which is accompanied by the reduction of genetic information.^147^ We, therefore, speculate that viruses may have originally been autonomous agents that later became intracellular parasites, which, however, cannot be proven.^33^

It appears that symbiogenesis of viruses is not a rare phenomenon either. Viral endosymbionts have been described, for instance, in insects such as *Drosophila melanogaster*.^148^ Insects are frequently infected with sigma viruses and *D. melanogaster* has evolved several mechanisms of resistance against the endosymbiotic viruses, yet the putative benefits to the host remain unclear.^148^ Interestingly, sigma viruses are likely purely vertically transmitted, indicating an adaptation to an endosymbiotic lifestyle and the loss of the ability of horizontal transmission.^148^ One example of how endosymbiotic viruses can be beneficial to their host, parasitoid wasps that lay eggs into other insects, is polydnaviruses.^139^ The viruses carry unusual genomes with characteristics of eukaryotic genes and protect the wasp's larvae from immune reactions by their insect host.^139^

Viral endosymbionts may also influence reproduction as in the case of an RNA virus found in the moth *Homona magnanima* that appears to be associated with late male killing.^149^ It has been suggested that viral endosymbionts may be an important but largely overlooked force in host evolution that require further investigation.^148^

## Conclusions

One can speculate that life on Earth started with the smallest known autonomously self‐replicating catalytically active entities, the ribozymes, which consist of noncoding circRNAs with information solely based on structure. Especially, the ribosomes suggest that ribozymes preceded prokaryotes because they supply the protein synthesis machinery in all forms of cellular life.^47^ Possibly, viruses, or more specifically viroid‐like agents, may have been among the first biological entities. Viruses may have started as simple, autonomously replicating elements, which after the emergence of cell‐like containments at the beginning of life lost genes to become obligate intracellular parasites. This kind of gene reduction is a frequent but often ignored evolutionary mechanism.^147^ Gene reduction was demonstrated *in vitro* by “Spiegelman's Monster,” where a long coding phage RNA in the presence of an RNA polymerase, free nucleotides, and salts degenerated to a small extremely fast replicating ncRNA after 74 generations, demonstrating that loss of genes for the benefit of fast replication can occur.^150^, ^151^ *Rickettsia*, mitochondria, and chloroplasts are other examples for loss of genes resulting in an exclusively intracellular lifestyle.^141^, ^142^, ^145^

Possibly, viruses gave up their independence for a parasitic lifestyle. In this case, viruses could be our oldest ancestors.^21^, ^33^ In this article, we sought to provide possible explanations as to how early ribozymes/viroids or virus‐like entities may have abandoned a former autonomy for a parasitic lifestyle, as observed today. Viruses are nowadays the most abundant biological entities on Earth, amounting to about 10^33^ particles.^152^ Noncoding RNAs, many of viral origin, have become major regulators of the coding genes in cellular organisms, which only constitute about 2% of mammalian genomes, whereas ncRNA represents about 98%.^1^, ^95^ The circular noncoding RNAs, circRNAs, are sponge‐like absorbing agents that regulate gene expression—they structurally resemble and might be evolutionarily related to the evolutionarily ancient ribozymes and viroids.^54^, ^55^ The importance of viruses and parasitic elements during genome evolution and antiviral responses has been pointed out by Villarreal before.^19^, ^20^, ^37^

There are some informative intermediates combining ncRNA and coding RNA, such as the group II introns that have taken up a reverse transcriptase gene.^116^ This is an important step of evolution, the transition from ncRNA to partially coding sequences—which may have led to the evolution of retroviruses.^50^

Recently, small ncRNA was identified as a genetic factor transmitted via sperm cells as a transgenerational carrier.^132^ Future studies will have to assess how frequent this phenomenon is. The similarities between viruses and phages as discussed here with respect to antiviral defense, shown for HERVs and CRISPR, demonstrate the close relatedness of the prokaryotic and the eukaryotic worlds.^18^ These similarities have often been overlooked. We may be able to compare the two seemingly distant worlds and find more similarities with respect to viruses, antiviral defense, viral counter‐defense, and others.

## Competing interests

The authors declare no competing interests.
